# Human-Like Receptor Specificity Does Not Affect the Neuraminidase-Inhibitor Susceptibility of H5N1 Influenza Viruses

**DOI:** 10.1371/journal.ppat.1000043

**Published:** 2008-04-11

**Authors:** Natalia A. Ilyushina, Elena A. Govorkova, Thomas E. Gray, Nicolai V. Bovin, Robert G. Webster

**Affiliations:** 1 Division of Virology, Department of Infectious Diseases, St. Jude Children's Research Hospital, Memphis, Tennessee, United States of America; 2 Laboratory of Virus Physiology, The D.I. Ivanovsky Institute of Virology RAMS, Moscow, Russia; 3 Laboratory of Molecular Carcinogenesis, National Institutes of Environmental Health Sciences, Research Triangle Park, North Carolina, United States of America; 4 Laboratory of Carbohydrate Chemistry, Shemyakin Institute of Bioorganic Chemistry, Moscow, Russia; 5 Department of Pathology, University of Tennessee Health Science Center, Memphis, Tennessee, United States of America; Erasmus Medical Center, The Netherlands

## Abstract

If highly pathogenic H5N1 influenza viruses acquire affinity for human rather than avian respiratory epithelium, will their susceptibility to neuraminidase (NA) inhibitors (the likely first line of defense against an influenza pandemic) change as well? Adequate pandemic preparedness requires that this question be answered. We generated and tested 31 recombinants of A/Vietnam/1203/04 (H5N1) influenza virus carrying single, double, or triple mutations located within or near the receptor binding site in the hemagglutinin (HA) glycoprotein that alter H5 HA binding affinity or specificity. To gain insight into how combinations of HA and NA mutations can affect the sensitivity of H5N1 virus to NA inhibitors, we also rescued viruses carrying the HA changes together with the H274Y NA substitution, which was reported to confer resistance to the NA inhibitor oseltamivir. Twenty viruses were genetically stable. The triple N158S/Q226L/N248D HA mutation (which eliminates a glycosylation site at position 158) caused a switch from avian to human receptor specificity. In cultures of differentiated human airway epithelial (NHBE) cells, which provide an *ex vivo* model that recapitulates the receptors in the human respiratory tract, none of the HA-mutant recombinants showed reduced susceptibility to antiviral drugs (oseltamivir or zanamivir). This finding was consistent with the results of NA enzyme inhibition assay, which appears to predict influenza virus susceptibility *in vivo*. Therefore, acquisition of human-like receptor specificity does not affect susceptibility to NA inhibitors. Sequence analysis of the NA gene alone, rather than analysis of both the NA and HA genes, and phenotypic assays in NHBE cells are likely to adequately identify drug-resistant H5N1 variants isolated from humans during an outbreak.

## Author Summary

If the avian influenza H5N1 viruses adapt to human hosts, the first step is likely to be a switch in the preference of their viral hemagglutinin (HA) glycoprotein to bind to human rather than avian cell receptors. Such a switch may also alter virus susceptibility to neuraminidase (NA) inhibitors, which are anti-influenza drugs that are likely to be the first line of defense against a pandemic. We generated recombinant A/Vietnam/1203/04-like (H5N1) viruses carrying HA mutations previously shown to alter receptor specificity or affinity. We also discovered a previously unknown route (three simultaneous HA amino acid substitutions) by which highly pathogenic H5N1 viruses can adapt to human receptors. We then used a novel cell-culture–based system (differentiated human airway epithelial NHBE cells) to evaluate the recombinant viruses' resistance to NA inhibitors. None of the HA-mutant recombinants showed reduced drug susceptibility. Our results indicate that the tested HA mutations are unlikely to cause resistance to NA inhibitors *in vivo*. The NHBE system meets the need for an appropriate cell-culture–based system for phenotypic characterization of drug resistance.

## Introduction

The spread of highly pathogenic avian influenza A (H5N1) viruses from Asia to the Middle East, Europe, and Africa raises serious concern about a potential human pandemic [1],[2]. H5N1 avian influenza virus has been reported in poultry in 63 countries; 359 human cases have been confirmed in 14 countries, with a mortality rate >60% [3]. A poor fit between avian viruses and human cellular receptors is thought to be one of the main barriers to efficient transmission of H5N1 influenza viruses between humans [2], [4]–[6]. The hemagglutinin (HA) glycoproteins of avian influenza viruses bind to avian cell-surface receptors whose saccharides terminate in sialic acid (SA)-α2,3-galactose (SAα2,3Gal), whereas those of human influenza viruses bind to human receptors whose saccharides end in SAα2,6Gal. A change in receptor specificity from SAα2,3Gal to SAα2,6Gal is thought to be necessary before avian influenza viruses can cause a pandemic [4]–[6].

Neuraminidase (NA) inhibitors (oseltamivir and zanamivir) are anti-influenza drugs that are likely to be the first line of defense in the event of an influenza pandemic, before antigenically matched influenza vaccine is available [1], [7]–[10]. Although HA mutations that alter viral receptor affinity/specificity can contribute to NA inhibitor resistance *in vitro* by allowing efficient virus release from infected cells without the need for significant NA activity [9], [11]–[18], the importance of HA mutations in the clinical management of influenza in humans remains uncertain [11], [19]–[23]. One important problem is the lack of a reliable experimental approach (i.e., an appropriate cell-culture–based system) for screening viral isolates for drug sensitivity [9],[11],[19],[20]. HA mutations can either increase or mask NA inhibitor resistance in the available assay systems, which are therefore susceptible to false-positive [24],[25] and false-negative [21],[22] results. This problem is likely to reflect a mismatch between human virus receptors and those in available cell-culture systems. The human airway epithelial cells targeted by influenza virus express high concentrations of SAα2,6Gal-containing receptors, which are present at low concentrations in the continuous cell lines used to propagate influenza viruses [9],[11],[19],[20],[26].

To test whether altered receptor-binding properties of the viral HA glycoprotein of highly pathogenic A/Vietnam/1203/04 (H5N1) influenza virus can reduce susceptibility to NA inhibitors *in vivo*, we generated 31 recombinant viruses carrying amino acid changes within or near the receptor binding site that alter binding affinity or specificity [27]. To evaluate the recombinant viruses' resistance to NA inhibitors, we used, for the first time, a cell-culture–based system that morphologically and functionally recapitulates differentiated human airway epithelial cells *ex vivo* [28],[29]. Based on our analysis, we propose that the HA mutations would not be expected to mediate resistance of H5N1 viruses to antiviral drugs, oseltamivir or zanamivir.

## Results

### Identification of HA Mutations that Alter the Receptor Specificity of A/Vietnam/1203/04 (H5N1) Influenza Virus

To test the hypothesis that substitutions in the viral HA gene can contribute to NA inhibitor resistance, we generated recombinant H5N1 viruses harboring HA point mutations that alter viral receptor specificity or affinity to SA receptors, using two approaches. Our group and others [11]–[18],[30],[31] had previously identified a number of HA mutations within and near the receptor binding site that could alter receptor specificity or affinity. However, as a first step in this study, we wished to identify additional HA point mutations that could convert the avian H5 HA to human-type receptor specificity. Previous studies had shown that two HA substitutions (Q226L and G228S) are likely to modulate receptor specificity in the H5 serotype [5]. We therefore passaged the wild-type virus (rgVN1203) and two HA mutants (Q226L and G228S) in MDCK-SIAT1 cells (Madin Darby canine kidney cells altered to express predominantly human-type SAα2,6 receptors). Because of the ability of NA inhibitors to select mutants with altered receptor affinity/specificity during *in vitro* passage, we also cultured these three H5N1 viruses in MDCK-SIAT1 cells in the presence of 1 µM oseltamivir [12]–[18]. Interestingly, infection with the wild-type virus was undetectable by PCR analysis after two passages with 1 µM of the NA inhibitor in two independent experiments (data not shown). Sequence analysis of the entire HA and NA genes revealed no additional mutations in virus with the G228S substitution after five sequential passages in the presence or absence of the drug. However, virus with the Q226L substitution had acquired two additional HA mutations, N158S (which eliminates a glycosylation site at position 158 [32]) and N248D, after five passages with or without compound. The receptor specificity of this triple-mutant (N158S/Q226L/N248D) virus was determined by measuring its binding affinity to sialoglycopolymers possessing either SAα2,3Gal (p3′SL) or SAα2,6Gal (p6′SL) (Table S1). This H5N1 variant exhibited enhanced affinity for human-like SAα2,6-linked receptor and was unable to bind the avian-like SAα2,3-linked receptor (Figure S1); therefore, the N158S/Q226L/N248D triple mutation is sufficient to completely switch the host receptor specificity of A/Vietnam/1203/04 (H5N1) virus from avian to human.

### Characterization of Recombinant A/Vietnam/1203/04 (H5N1) Viruses with HA Mutations in or near the Receptor Binding Site That Alter Receptor Specificity or Affinity

**Figure 1:**
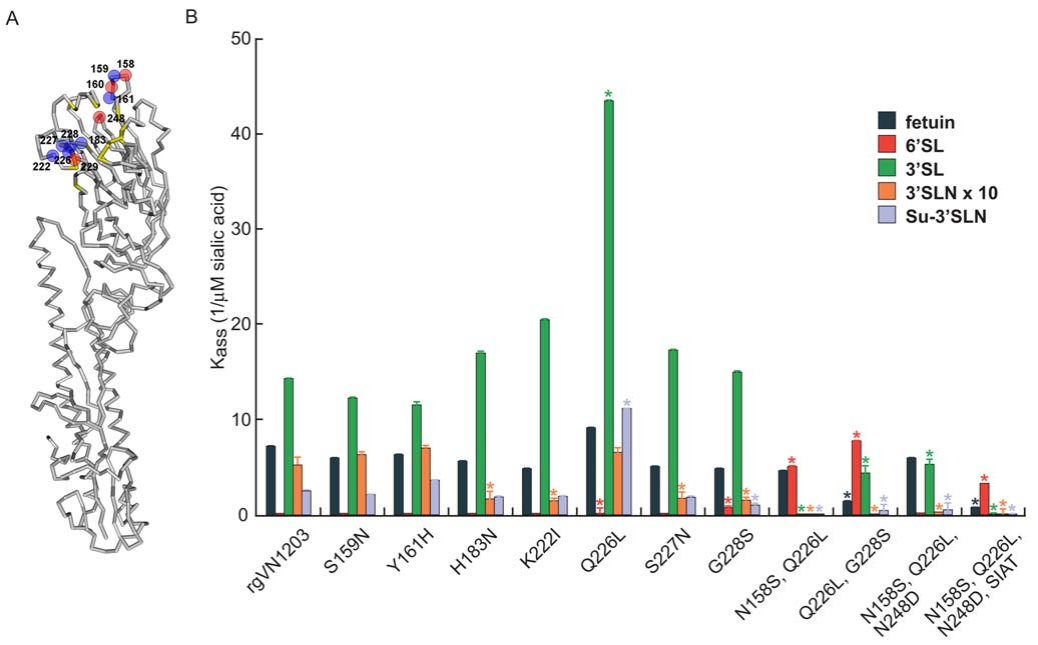
(A) Crystal structure of the A/Vietnam/1203/04 hemagglutinin molecule (Protein Data Bank:2FK0) showing 11 positions in or near the receptor binding site (yellow) at which amino acid substitutions can alter receptor specificity/affinity. Residues in red are positions where a single substitution affects the stability of the H5N1 virus. (B) Affinity of recombinant A/Vietnam/1203/04-like (H5N1) influenza A viruses for high molecular weight sialic acid substrates, both natural (fetuin) and synthetic. Of the nine substrates tested (Table S1), only the five shown here had a K_ass_ significantly different from that of the negative control. Bars represent the association constants (K_ass_) of virus in complex with sialylglycopolymer. Values are the means±s.d. of four independent experiments.

Our second approach was to use reverse genetics [33] to generate recombinant A/Vietnam/1203/04-like (H5N1) viruses carrying HA mutations previously shown to alter receptor specificity or affinity [11]–[18],[30],[31]. This study characterized a total of 15 HA mutants (Table 1) carrying substitutions at a total of 11 positions (Figure 1A). In addition, to gain insight into how combinations of HA and NA mutations can affect the sensitivity of H5N1 virus to NA inhibitors, we rescued viruses carrying the 15 HA changes together with the H274Y NA substitution. This mutation is most frequently associated with the resistance to the NA inhibitor oseltamivir in the N1 NA subtype [11] and was extensively characterized in A/Vietnam/1203/04 (H5N1)-virus background both *in vitro* and *in vivo* [34] (Table S2).

**Table 1:** Hemagglutinin amino acid substitutions in A/Vietnam/1203/04-like (H5N1) recombinant viruses generated in this study

No. of HA amino acid substitutions	Specific substitutions^a^	Location of substitutions in relation to receptor-binding site
**One**	N158S	form a N-linked glycosylation site at position 158, atop and to the side of the receptor pocket
	S159N	
	T160A	
	Y161H	next to glycan at position 158 closed to the receptor pocket
	H183N	forms the rear of the site via a chain of hydrogen bonds linking W153, Y195, Y98, and links via a water molecule to E190
	K222I	near the left edge of receptor pocket
	Q226L	form the left edge of receptor pocket
	S227N	
	G228S	
	R229S	next to the left edge of receptor pocket
**Two**	N158S/Q226L	N158 forms glycan closed to the site and Q226 forms the left edge of the receptor pocket
	N158S/N248D	N158 forms glycan closed to the site and N248 is located in the upper part of the HA globular head close to the edge of the receptor pocket
	Q226L/G228S	form the left edge of receptor pocket
	Q226L/N248D	Q226 forms the left edge of the receptor pocket and N248 is located in the upper part of the HA globular head
**Three**	N158S/Q226L/N248D	N158 forms glycan closed to the site; Q226 forms the left edge of the receptor pocket; N248 is located in the upper part of the HA globular head close to the edge of the receptor pocket

a Amino acid numbering is based on H3 HA (Nobusawa et al., 1991, Virology, 182, 475–485).

Note: Underlined HA mutations caused an unstable phenotype. The genetic stability of recombinant H5N1 viruses was monitored by plaque assay and by sequencing of the HA and NA genes after transfection and after one passage in MDCK cells. Influenza virus was defined as genetically stable if it was able to replicate efficiently in the cell line used, maintain a homogeneous plaque phenotype, and did not contain additional subpopulations based on the sequence analysis of the HA and NA genes after one passage in MDCK cells. If different subpopulations were identified, those viruses were designated as unstable.

The use of the eight-plasmid reverse genetics system allowed us to predict the viability of all 31 recombinant H5N1 viruses in nature. We were able to rescue all of the recombinant viruses from transfected 293T cells as described previously [33]. However, the introduced N158S, T160A, R229S, N158S/N248D, and Q226L/N248D HA mutations could not be stably maintained in A/Vietnam/1203/04 virus after one passage in MDCK cells in two independent experiments because additional HA mutations were observed (Table S2). Interestingly, A/Vietnam/1203/04 virus simultaneously carrying the Q226L HA mutation and the H274Y NA mutation was genetically unstable, since the stock virus contained a mixture of viruses with Q or L at HA residue 226 as well as a K222I HA substitution. Sequence analysis revealed that the remaining 20 recombinant H5N1 viruses were stably maintained in stock cultures; these viruses grew to comparable titers in the different cell lines used (Table S2).

We measured the affinity of the 21 genetically stable recombinant H5N1 variants, including wild-type virus, for a wide range of high-molecular-weight sialic acid substrates, both natural (fetuin) and synthetic (Table S1). Like most avian influenza strains, wild-type rgVN1203 virus showed a binding preference for avian SAα2,3Gal-receptors (Figure 1B). The introduced HA substitutions had various effects on the receptor binding affinity of the H5 hemagglutinin to one or several SAα2,3Gal-substrates (Figure 1B). Surprisingly, H5N1 mutants carrying the triple mutation N158S/Q226L/N248D exhibited very weak SAα2,6Gal binding, whereas virus with the double mutation N158S/Q226L did not bind to any SAα2,3Gal sialosides but showed enhanced binding affinity to SAα2,6Gal-substrate (Figure 1B).

After observing a discrepancy in the receptor affinity of two N158S/Q226L/N248D HA triple mutants that were independently obtained by passaging in MDCK-SIAT1 cells (Figure S1) or by transfection of 293T-MDCK cells (Figure 1B), we investigated whether the host cell type can determine viral binding properties. We prepared virus stocks by transfecting MDCK-SIAT1 cells, rather than MDCK cells, with the two H5N1 viruses carrying the double N158S/Q226L and triple N158S/Q226L/N248D HA substitutions. Direct sequencing of the double-mutant virus revealed the presence of additional mutations; therefore, we did not assay its receptor specificity. A/Vietnam/1203/04 (H5N1) virus carrying the triple N158S/Q226L/N248D HA mutation and grown in MDCK-SIAT1 cells was genetically stable and demonstrated the switch from avian to human receptor specificity (Figure 1B).

### Susceptibility of the Recombinant H5N1 Viruses to NA Inhibitors

We used the fluorometric NA enzyme inhibition assay [35] to test the susceptibility to oseltamivir and zanamivir of the 20 recombinant H5N1 viruses carrying either HA mutations or both HA and NA (H274Y) changes. None of the recombinants carrying only HA mutations differed from the wild-type virus in their sensitivity to either NA inhibitor (mean IC_50_±SD, 0.2±0.1 and 1.0±0.1 nM, respectively, for oseltamivir and zanamivir). All double-gene–mutant recombinant viruses were resistant to oseltamivir (the mean IC_50_ was ∼2060 times that of wild-type virus) but remained highly susceptible to zanamivir (IC_50_, 1.0±0.2 nM) (data not shown).

**Figure 2:**
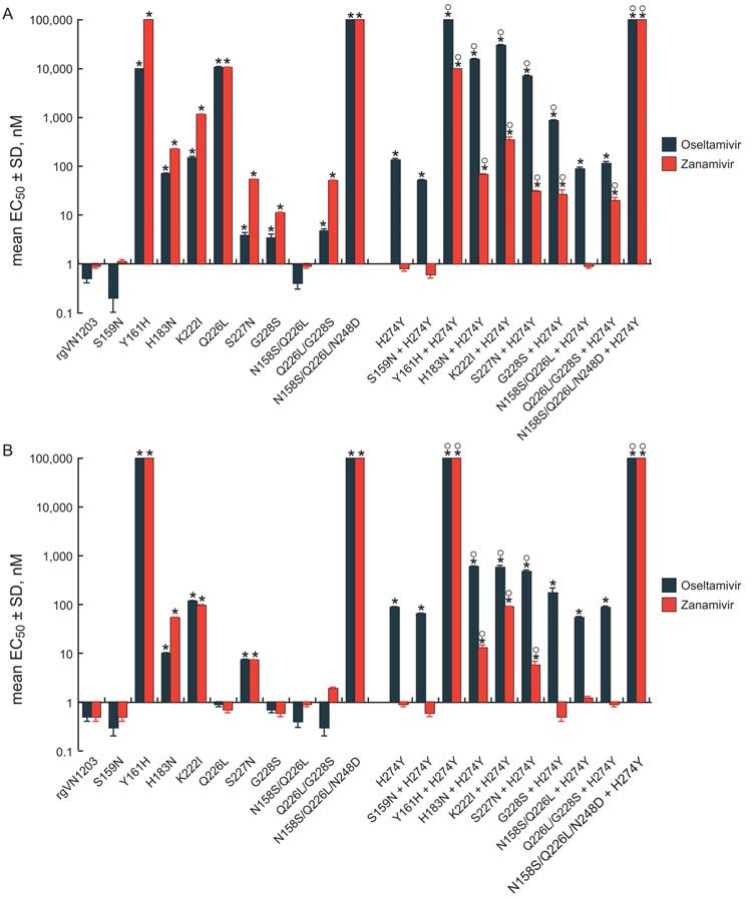
Susceptibility of recombinant A/Vietnam/1203/04-like (H5N1) influenza A viruses to NA inhibitors in (A) MDCK cells and (B) MDCK-SIAT1 cells as determined by plaque reduction assay. EC_50_ was the dose of drug required to reduce plaque size by 50%. Values are the means±s.d. of three independent experiments. * *P*<0.01 compared to wild-type rgVN1203 virus (one-way ANOVA performed for all viruses); °*P*<0.01 compared to virus carrying only the NA H274Y substitution (one-way ANOVA performed for viruses carrying the H274Y NA mutation).

**Figure 3:**
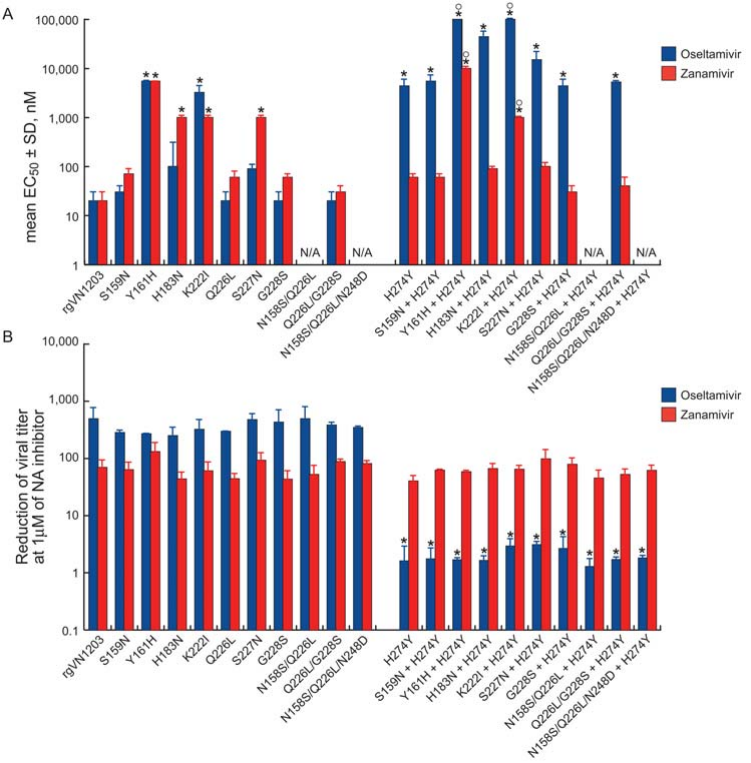
Susceptibility of recombinant A/Vietnam/1203/04-like (H5N1) influenza A viruses to NA inhibitors in (A) A549 and (B) NHBE cells as determined by virus reduction assay. In A549 cells, EC_50_ was the dose of drug that reduced the TCID_50_ titer of culture supernatant 50% as compared to no-drug controls 72 h after infection. In NHBE cells, virus yield was measured after 24 h incubation with 0, 0.1, 1, or 10 µM of NA inhibitor by TCID_50_ assay in MDCK cells. All concentrations yielded similar results; those obtained with 1 µM NA inhibitor are shown. * *P*<0.01 compared to wild-type rgVN1203 virus, one-way ANOVA test performed for all viruses; °*P*<0.01 compared to H274Y virus, one-way ANOVA test performed for viruses carrying the H274Y NA mutation.

We next compared the activity of NA inhibitors in four cell-culture systems that differ in the surface distribution of SAα2,3- and SAα2,6-receptors. We performed plaque reduction assays in MDCK and MDCK-SIAT1 cells and virus reduction assays in human alveolar basal epithelial (A549) and normal human bronchial epithelial (NHBE) cells (Figures 2 and 3).

In MDCK cells, which express predominantly SAα2,3 (avian-type) receptors [26], all HA mutants except those with the S159N and N158S/Q226L substitutions were significantly more resistant to oseltamivir and to zanamivir than the wild-type strain (*P*<0.01) (Figure 2A). Introduction of the NA H274Y mutation markedly reduced the sensitivity of the recombinant H5N1 viruses to oseltamivir (mean EC_50_, 270 times that of the wild-type strain) but did not alter their susceptibility to zanamivir. The combined HA and NA (H274Y) substitutions therefore reduced oseltamivir sensitivity synergistically in MDCK cells.

In MDCK-SIAT1 cells, which have predominantly SAα2,6 (human-type) receptors [26], most of the mutants showed resistance to both NA inhibitors (Figure 2B), but fewer viruses were resistant in MDCK-SIAT1 than in MDCK cells. Viruses with Q226L, G228S and Q226L/G228S substitutions, which enhanced binding affinity for SAα2,6Gal receptors (Figure 1B), were as sensitive to both NA inhibitors as the wild-type virus in MDCK-SIAT1 cells but not in MDCK cells (Figure 2). Taken together, our results indicated that the drug sensitivity of the recombinant H5N1 viruses detected in MDCK-SIAT1 cells reflected their affinity for SAα2,6Gal rather than for SAα2,3Gal receptors.

In human lung A549 cells, which have predominantly SAα2,6-receptors [36], the overall sensitivity pattern was similar to that observed in MDCK-SIAT1 cells (Figure 3A). However, we were unable to assay the drug susceptibility of the mutants carrying the double N158S/Q226L and triple N158S/Q226L/N248D HA mutations, because their replication was undetectable. In A549 cells, but not in MDCK and MDCK-SIAT1 cells, only double mutants with the Y161H or K222I HA substitution plus the H274Y NA mutation showed resistance significantly greater (EC_50_ increased by a factor of 10-10^6^) than that of the H274Y virus to both NA inhibitors (Figure 3A).

In differentiated cultures of NHBE cells (primarily human-type SAα2,6-receptors [28]), none of the HA mutations resulted in increased resistance to oseltamivir or zanamivir, and there was no difference in susceptibility between viruses carrying only the H274Y NA mutation and those carrying HA mutations as well (Figure 3B). Therefore, the combined HA–NA mutations had a negligible effect on the NA inhibitor sensitivity of H5N1 viruses in NHBE cultures. All recombinant viruses carrying a single HA substitution were slightly less susceptible to zanamivir than to oseltamivir (the EC_50_ differed by a factor of ∼5) in NHBE cells. This finding was consistent with the data obtained by NA enzyme inhibition assay (see above).

## Discussion

Our results answer several fundamental questions about the effect of HA mutations on the host receptor affinity and NA inhibitor susceptibility of highly pathogenic influenza H5N1 viruses. Importantly, we found that alteration of receptor specificity or affinity does not alter sensitivity to NA inhibitors. In light of global concern about pandemic preparedness, it is crucial not only to understand what mutations might endow H5N1 viruses with human receptor specificity [4]–[6] but also to anticipate the clinical consequences of such adaptation. With increasing clinical use and stockpiling of NA inhibitors for pandemic preparedness, it is also crucial to elucidate molecular mechanisms that contribute to drug resistance.

Mutations at specific positions (129/134, 182, 192, 227, and 226/228) in the HA gene of H5N1 influenza virus were recently shown to reduce or eliminate binding affinity to avian-type receptors and enhance affinity to human-type receptors [5],[6],[37],[38]. Here, we have identified another possible route of adaptation to human receptors: simultaneous amino acid substitutions at HA positions 158 (that results in the loss of a glycosylation site), 226 and 248. Our study demonstrated the importance not only of residues near or within the receptor binding site, but also those structural elements that are located in its vicinity that can affect host receptor specificity. H5N1 influenza viruses in H5 clade 1, such as the virus used here, would be extremely unlikely to acquire all three mutations. However, most members of the clade 2.2 family now circulating in Europe, the Middle East and Africa already lack a glycosylation site at HA position 158 [1]. This natural feature reduces the required mutations to only two, thus enhancing the probability of such an occurrence. Taken together, we can conclude that different amino acid substitutions in the H5 HA enable to cause a shift from the avian- to human-type specificity and along with the H5N1 evolution other strain-specific mutations cannot be excluded.

HA glycosylation has been reported to affect the specificity or affinity of influenza viruses for cellular receptors [39]–[41]. Our results directly demonstrate that HA glycosylation plays a role in the viability of avian H5N1 influenza viruses in MDCK-SIAT1 cells, which express predominantly human-like SAα2,6Gal receptors. We demonstrated that the loss of an MDCK-SIAT1 cell–synthesized oligosaccharide at position 158 of HA, adjacent to the receptor binding site, increased the capacity of the virus to bind to these cells. One possible explanation is that the removal of an oligosaccharide attachment site from the tip of the HA1 subunit eliminates steric hindrance that limits the accessibility of the receptor pocket by this SA-containing oligosaccharide [41]. Further, our results confirm previous reports [39]–[41] that the effect of oligosaccharide removal can differ with the host cell type (MDCK vs. MDCK-SIAT1) in which the H5N1 virus is grown. Therefore, the receptor affinity of the avian H5N1 influenza virus may also be affected by the host cell–determined composition of other oligosaccharides on the two H5 HA subunits.

Our results are consistent with what is known about the role of the functional balance between the receptor-binding (HA) and receptor-destroying (NA) activities of the surface glycoproteins in efficient influenza virus infection [42],[43]. The genetic and phenotypic instability of 11 of our recombinant H5N1 viruses *in vitro* may reflect the functional mismatch of their HA and NA glycoproteins. The balance between HA and NA functions could also explain the diverse pattern of influenza virus susceptibility to NA inhibitors observed in different cell-culture systems [11],[19],[20],[42],[43]. The disparate HA–NA balance required to infect MDCK, MDCK-SIAT1, and A549 cells, together with the differences in SA receptors between these cell lines and human respiratory epithelial cells, significantly limit the suitability of these commonly used cell lines for phenotypic characterization of NA inhibitor resistance.

NHBE cells cultured *ex vivo* [28],[29],[44] offer a new cell-culture–based system that functionally and morphologically recapitulates normal differentiated human airway epithelium; this system allows improved evaluation of the NA inhibitor sensitivity of avian influenza viruses that are potential human pathogens. Taken together, our data demonstrate a parallel between virus susceptibility determined by NA enzyme inhibition assay (which appears to predict *in vivo* results [11],[19],[20]) and virus susceptibility in NHBE cells (an *ex vivo* model). NHBE cells [28],[44], which express the sialic acid receptors present in humans, may offer an optimal system for maintaining viral fitness and, as a consequence, for prediction of influenza virus resistance to NA inhibitors *in vivo*. Our results suggest that the HA mutations that alter the receptor specificity or affinity of highly pathogenic H5N1 viruses are unlikely to mediate concomitant resistance to NA inhibitors *in vivo*.

However, we cannot exclude the possibility that the HA mutations might contribute to the selection of certain NA mutations that lead to drug resistance simply by altering HA–NA balance. Indeed, recent observation that H5N1 viruses from clade 2 isolated in 2005 demonstrated a 25- to 30-fold decrease in sensitivity to oseltamivir carboxylate compared with clade 1 viruses and none of the mutations known to confer NA inhibitor resistance was observed [45], suggests that the decrease in sensitivities may be due to drift mutations in the NA and HA proteins. Additionally, our finding that A/Vietnam/1203/04 virus carrying the Q226L HA mutation, which is known to switch the receptor specificity in the H3 HA subtype [5], and the H274Y NA mutation was not genetically stable, could provide evidence that some HA mutations have the potential impact on the acquisition of mutations in NA, including those that can lead to decreased drug susceptibility. One, therefore, can speculate that the identification of oseltamivir-resistant viruses as a significant proportion of influenza H1N1 viruses circulating in Europe [46] could be determined by preceding NA and/or HA mutations.

In conclusion, our findings can improve the monitoring of NA inhibitor resistance among viruses with pandemic potential. Further, sequence analysis of the NA gene alone, rather than analysis of both the NA and HA genes, may adequately identify all drug-resistant H5N1 variants. The human airway epithelial cell cultures used in this study could also advance the study of drug resistance mechanisms by serving as a suitable model of the human respiratory cell system for phenotypic characterization of NA inhibitor resistance in clinical testing.

## Materials and Methods

### Cells, viruses and compounds

Madin-Darby canine kidney (MDCK), human embryonic kidney (293T) and human alveolar basal epithelial (A549) cells were obtained from the American Type Culture Collection. MDCK cells transfected with cDNA encoding human 2,6-sialyltransferase (MDCK-SIAT1 cells) were kindly provided by Dr. Mikhail N. Matrosovich. Primary normal human bronchial epithelial (NHBE) cells were obtained from Cambrex Bio Science. All cell cultures were maintained as previously described [26],[28],[31],[36],[44].

Eight plasmids were constructed from the DNA sequences of the 8 gene segments of wild-type A/Vietnam/1203/04 (H5N1) virus for the reverse-genetics generation of recombinant wild-type virus (rgVN1203). Recombinant virus was generated by DNA transfection of 293T cells [33], the HA cleavage site was removed, and the point mutations (Tables 1 and S2, Figure 1A) were inserted into the HA and NA genes of rgVN1203 virus by using a Quickchange site-directed mutagenesis kit (Stratagene) [32]. Stock viruses were prepared in MDCK cells at 37°C for 72 h and their entire HA and NA genes were sequenced to verify the presence of the mutations. The recombinant viruses were designated according to their HA and NA mutations (Tables 1 and S2). All experimental work with the H5N1 recombinant viruses was performed in a biosafety level 3+ laboratory approved for use by the U.S. Department of Agriculture and the U.S. Centers for Disease Control and Prevention.

The NA inhibitors oseltamivir carboxylate (oseltamivir) ([*3R,4R,5S*]-4-acetamido-5-amino-3-[1-ethylpropoxy]-1-cyclohexene-1-carboxylic acid) and zanamivir (4-guanidino-Neu5Ac2en) were provided by Hoffmann-La Roche, Ltd.

### Cultivation of H5N1 influenza viruses in the presence or absence of oseltamivir for identification of HA mutations that alter receptor specificity

For the first passage, MDCK-SIAT1 cells were infected with influenza H5N1 viruses at a multiplicity of infection (MOI) of 0.001 PFU/cell and cultivated for 72 h in infection medium [containing 4% bovine serum albumin, sodium bicarbonate, 100 U/ml of penicillin, 100 µg/ml of streptomycin sulfate, 100 µg/ml of kanamycin sulfate, 1 µg/ml of L-1-(tosyl-amido-2-phenyl)ethyl chloromethyl ketone (TPCK)–treated trypsin (trypsin) (Worthington Diagnostics)] with or without 1 μM oseltamivir [31]. Four additional passages identical to the first one were then performed sequentially.

### Stability and infectivity of recombinant H5N1 viruses

The genetic stability of recombinant H5N1 viruses was monitored by plaque assay and by sequencing of the HA and NA genes after transfection of 293T cells and after one passage in MDCK/MDCK-SIAT1 cells. Influenza virus was defined as genetically stable if it was able to replicate efficiently in the cell lines used, maintain a homogeneous plaque phenotype, and did not contain additional subpopulations based on the sequence analysis of the HA and NA genes after one passage in MDCK/MDCK-SIAT1 cells. If different subpopulations were identified, those viruses were designated as unstable (Tables 1 and S2).

The yield of H5N1 viruses in MDCK, MDCK-SIAT1 and A549 cells was defined as log_10_ of the 50% tissue culture infectious dose (TCID_50_) as described previously [43]. Briefly, confluent monolayers of cell cultures growing in 96-well microplates were inoculated with serial virus dilutions (each dilution was added to five wells) in the presence of trypsin. After 3 days, virus was titrated by HA assay, and virus titers were expressed as log_10_TCID_50_/ml by the end-point method of Reed and Muench [47]. NHBE cells were inoculated by exposure of the apical side to recombinant H5N1 viruses at a MOI of 0.1, as determined by TCID_50_ assay in MDCK cells. After 1 h incubation, the inoculum was removed and the cells were incubated for 24 h. No trypsin was added to the cultures because previous studies in similar cultures demonstrated efficient proteolytic activation of influenza viruses by endogenous proteases [28]. Viruses released into the apical compartment of NHBE were harvested by adding 300 µl of medium to the apical compartment, allowing it to equilibrate for 30 min, and collecting it. Virus titer was determined as log_10_TCID_50_/ml in MDCK cells.

### Receptor-binding assay

The binding of human influenza viruses to fetuin was measured in a direct solid-phase assay using the immobilized virus and horseradish peroxidase-conjugated fetuin, as described previously [48]. The affinity of viruses for synthetic 3′- and 6′-substrates (Table S1) was measured in a competitive assay based on the inhibition of binding of the labeled fetuin [49]. The association constants (*K*_ass_) were determined as sialic acid (Neu5Ac) concentration at the point A_max_/2 on Scatchard plots.

### NA enzyme inhibition assay

NA activity was determined as described by Potier et al. [35]. Briefly, H5N1 viruses and various concentrations of oseltamivir or zanamivir were preincubated for 30 min at 37°C before addition of the substrate 2′-(4-methylumbelliferyl)-α-D-N-acetylneuraminic acid (Sigma). After 1 h, the reaction was terminated by adding 14 mM NaOH and fluorescence was quantified in a Perkin-Elmer fluorometer. The IC_50_ was defined as the concentration of NA inhibitor necessary to reduce NA activity by 50% relative to that in a reaction mixture containing virus but no inhibitor.

### Drug susceptibility assays

The drug susceptibility of recombinant H5N1 viruses was determined by plaque reduction assay in MDCK and MDCK-SIAT1 cells [50] and by virus reduction assay in A549 and NHBE cells [31],[51]. Briefly, MDCK or MDCK-SIAT1 cells were inoculated with virus diluted to yield ∼50 plaques per well and were then overlaid with infection medium containing oseltamivir (0.0001 to 100 µM) or zanamivir (0.0001 to 100 µM) in the presence of trypsin. The results were recorded after 3 days of incubation at 37°C. At least three independent experiments were performed to determine the concentration of compound required to reduce plaque size by 50%, relative to that in untreated wells (EC_50_).

A549 cells were inoculated with H5N1 viruses at an MOI of 0.001 PFU/cell and after 1 h of adsorption were overlaid with infection medium containing oseltamivir (0.001 to 100 µM) or zanamivir (0.001 to 100 µM) in the presence of trypsin. Virus yield was determined by TCID_50_ assay of culture supernatants 72 h after inoculation. The drug concentration that caused a 50% decrease in the TCID_50_ titer in comparison to control wells without drug was defined as the 50% inhibitory concentration (EC_50_). The results of three independent experiments were averaged.

NHBE cells were inoculated by exposure of the apical side to recombinant H5N1 viruses at an MOI of 0.1 in the presence of oseltamivir (0, 0.1, 1, 10 µM) or zanamivir (0, 0.1, 1, or 10 µM). These concentrations represent typical plasma minimum and maximum concentrations measured in humans after administration of 75 mg of oseltamivir phosphate or 10 mg of zanamivir, the doses recommended for prophylaxis [10],[52]. After 1 h incubation, the inoculum was removed and the cells were incubated for another 24 h. Viruses released into the apical compartment of NHBE cells were harvested by the apical addition and collection of 300 µl of medium allowed to equilibrate for 30 min. The virus titer was determined as log_10_TCID_50_/ml in MDCK cells.

## Supporting Information

Figure S1 Affinity of H5N1 variant with N158S/Q226L/N248D triple mutation isolated after five passages in MDCK-SIAT1 cells for sialyl substrates. Each data bar represents association constant (K_ass_) of virus in complex with sialylglycopolymer p3′SL or p6′SL (Table S1). Higher K_ass_ values indicate stronger binding. Values are the means±s.d. of four independent experiments.
(0.13 MB TIF)

Table S1 Synthetic oligosaccharide-polyacrylamide substrates used to test the receptor specificity of H5N1 viruses
(108 KB DOC)

Table S2 *In vitro* characterization of recombinant H5N1 viruses
(31.0 KB DOC)
