# Supplementary figures and images for: Human-Like Receptor Specificity Does Not Affect the Neuraminidase-Inhibitor Susceptibility of H5N1 Influenza Viruses
Source: PLoS Pathog. 2008 Apr 11;4(4):e1000043. doi: 10.1371/journal.ppat.1000043 (PMC2276691; doi:10.1371/journal.ppat.1000043)

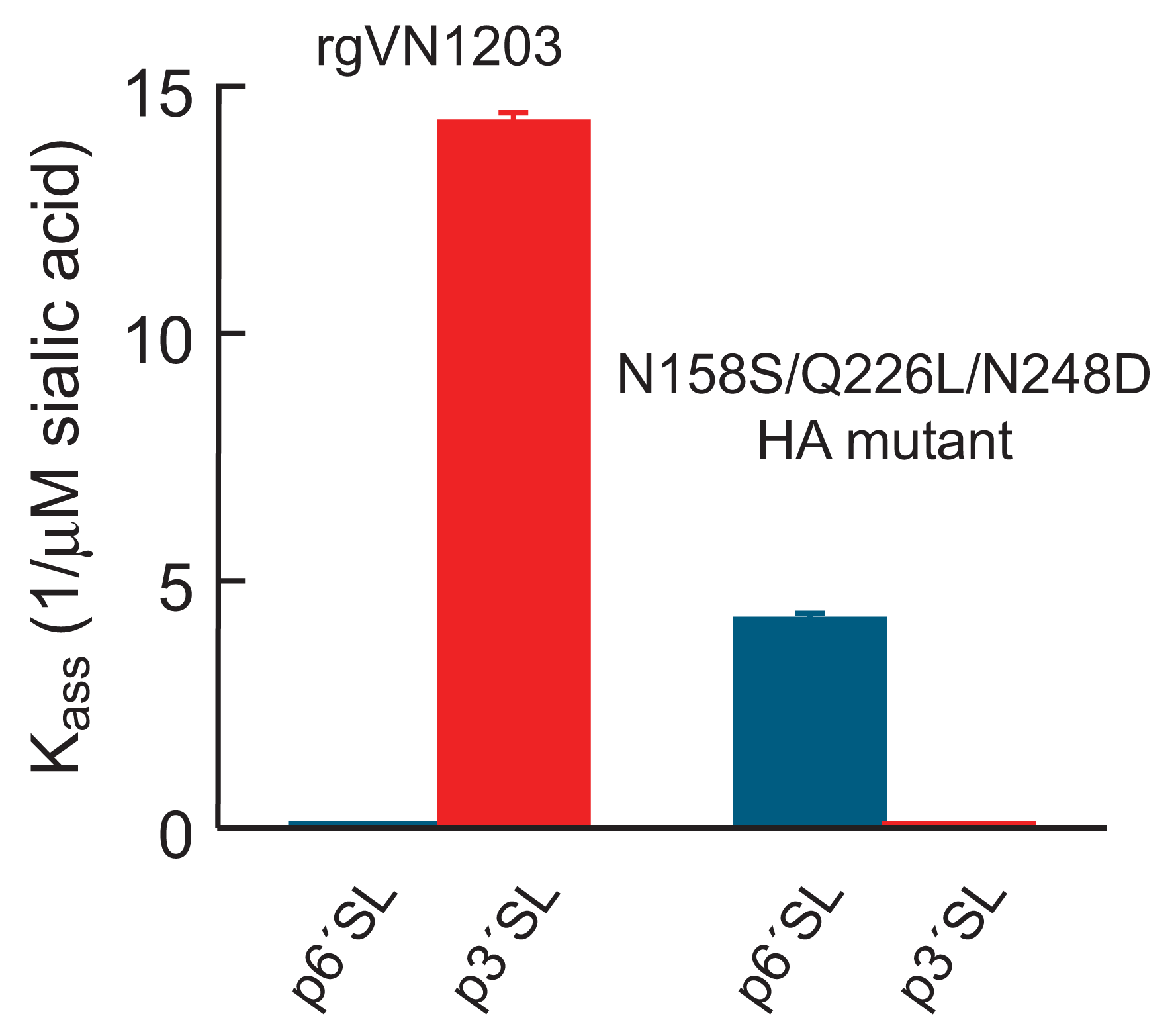

Supplement: Figure S1 — Affinity of H5N1 variant with N158S/Q226L/N248D triple mutation isolated after five passages in MDCK-SIAT1 cells for sialyl substrates. Each data bar represents association constant (Kass) of virus in complex with sialylglycopolymer p3′SL or p6′SL (Table S1). Higher Kass values indicate stronger binding. Values are the means±s.d. of four independent experiments. (0.13 MB TIF) [file ppat.1000043.s001.tif]
