# Supplementary material for: Human-Like Receptor Specificity Does Not Affect the Neuraminidase-Inhibitor Susceptibility of H5N1 Influenza Viruses
Source: PLoS Pathog. 2008 Apr 11;4(4):e1000043. doi: 10.1371/journal.ppat.1000043 (PMC2276691; doi:10.1371/journal.ppat.1000043)
Supplement: Table S1 — Synthetic oligosaccharide-polyacrylamide substrates used to test the receptor specificity of H5N1 viruses (108 KB DOC) [file ppat.1000043.s002.doc]

**Table S1.** Synthetic oligosaccharide-polyacrylamide substrates used to test the receptor

specificity of H5N1 viruses

| **Structure of oligosaccharide** | **Abbreviation** |
| --- | --- |
| Neu5Acα2–6Galβ1–4Glcβ | 6′SL |
| Neu5Acα2–6Galβ1–4GlcNAcβ | 6′SLN |
| Neu5Acα2–3Galβ1–4Glcβ | 3′SL |
| Neu5Acα2–3Galβ1–4GlcNAcβ | 3′SLN |
| Neu5Acα2–3Galβ1–4–(6–Su)GlcNAcβ | Su-3′SLN |
| Neu5Acα2–3Galβ1–3GlcNAcβ | SiaLe^C^ |
| Neu5Acα2–3Galβ1–4(Fucα1–3)GlcNAcβ | SiaLe^X^ |
| Neu5Acα2–3Galβ1–3(Fucα1–4)GlcNAcβ | SiaLe^A^ |

Sialoglycoconjugates with different inner saccharide residues were earlier used to characterize the receptor specificity of recombinant H5N1 viruses (Bovin et al., 1993, Glycoconj. J., 10, 142-151; Tuzikov et al., 2000, J. Carbohydr. Chem., 19, 1191-1200) and were used here in a competitive assay with peroxidase-labeled fetuin. SiaLe^C^ differs from 3′SLN by the type of linkage between galactose and the adjacent glucosamine (β1–3 in SiaLe^c^ and β1–4 in 3′SLN). SiaLe^X^ is 3′SLN fucosylated at O-3 of glucosamine. Sulfated saccharide (Su-3′SLN) carries the sulfo-group at O-6 of glucosamine.
