# Supplementary material for: Human-Like Receptor Specificity Does Not Affect the Neuraminidase-Inhibitor Susceptibility of H5N1 Influenza Viruses
Source: PLoS Pathog. 2008 Apr 11;4(4):e1000043. doi: 10.1371/journal.ppat.1000043 (PMC2276691; doi:10.1371/journal.ppat.1000043)
Supplement: Table S2 — In vitro characterization of recombinant H5N1 viruses (31.0 KB DOC) [file ppat.1000043.s003.doc]

**Table S2. *In vitro* characterization of recombinant H5N1 viruses**

| **Amino acid changes in the reverse genetics viruses^a^** | | **Genetic stability of viruses^b^** | **Viral growth in cell culture (log_10_TCID_50_/ml)** | | | |
| --- | --- | --- | --- | --- | --- | --- |
| **HA** | **NA** |  | **MDCK** | **MDCK-SIAT1** | **A549** | **NHBE** |
| – | – | stable | 7.6 ± 0.1 | 6.9 ± 0.2 | 6.7 ± 0.1 | 6.3 ± 0.3 |
| **N158S** | – | unstable | N/A | N/A | N/A | N/A |
| **S159N** | – | stable | 8.2 ± 0.1** | 7.9 ± 0.2** | 6.3 ± 0.3 | 5.8 ± 0.7 |
| **T160A** | – | unstable | N/A | N/A | N/A | N/A |
| **Y161H** | – | stable | 6.8 ± 0.1** | 7.3 ± 0.1 | 6.8 ± 0.3 | 6.2 ± 1.1 |
| **H183N** | – | stable | 7.9 ± 0.2 | 6.7 ± 0.5 | 7.1 ± 0.1 | 6.6 ± 0.9 |
| **K222I** | – | stable | 8.3 ± 0.2** | 6.9 ± 0.1 | 7.2 ± 0.4 | 7.6 ± 0.3** |
| **Q226L** | – | stable | 7.9 ± 0.1 | 7.1 ± 0.3 | 6.7 ± 0.1 | 7.3 ± 0.2* |
| **S227N** | – | stable | 8.0 ± 0.2 | 7.5 ± 0.3** | 7.5 ± 0.5** | 8.0 ± 0.7** |
| **G228S** | – | stable | 8.2 ± 0.2** | 7.2 ± 0.2 | 7.3 ± 0.5* | 7.2 ± 0.8 |
| **R229S** | – | unstable | N/A | N/A | N/A | N/A |
| **N158S/Q226L** | – | stable | 8.2 ± 0.1** | 8.0 ± 0.2** | 7.3 ± 0.4** | 8.2 ± 0.7** |
| **N158S/N248D** | – | unstable | N/A | N/A | N/A | N/A |
| **Q226L/G228S** | – | stable | 7.4 ± 0.6 | 6.6 ± 0.2 | 6.7 ± 0.1 | 7.8 ± 0.5** |
| **Q226L/N248D** | – | unstable | N/A | N/A | N/A | N/A |
| **N158S/Q226L/N248D** | – | stable | 8.1 ± 0.3* | 7.9 ± 0.2** | 6.9 ± 0.3 | 7.5 ± 1.0** |
| – | **H274Y** | stable | 6.9 ± 0.1** | 6.5 ± 0.1 | 6.0 ± 0.2** | 5.7 ± 0.2 |
| **N158S** | **H274Y** | unstable | N/A | N/A | N/A | N/A |
| **S159N** | **H274Y** | stable | 7.6 ± 0.1ºº | 7.6 ± 0.3**,ºº | 6.0 ± 0.2** | 5.4 ± 0.5 |
| **T160A** | **H274Y** | unstable | N/A | N/A | N/A | N/A |
| **Y161H** | **H274Y** | stable | 7.1 ± 0.1* | 7.5 ± 0.1*,ºº | 6.5 ± 0.2 | 6.4 ± 0.8 |
| **H183N** | **H274Y** | stable | 7.9 ± 0.3ºº | 6.3 ± 0.5** | 6.7 ± 0.2ºº | 6.8 ± 0.3º |
| **K222I** | **H274Y** | stable | 7.8 ± 0.1ºº | 7.2 ± 0.1ºº | 6.7 ± 0.1ºº | 6.7 ± 0.3º |
| **Q226L** | **H274Y** | unstable | N/A | N/A | N/A | N/A |
| **S227N** | **H274Y** | stable | 7.7 ± 0.5ºº | 7.7 ± 0.2**,ºº | 6.9 ± 0.1ºº | 7.2 ± 0.4ºº |
| **G228S** | **H274Y** | stable | 8.0 ± 0.2ºº | 6.9 ± 0.2 | 6.3 ± 0.1 | 7.2 ± 0.5ºº |
| **R229S** | **H274Y** | unstable | N/A | N/A | N/A | N/A |
| **N158S/Q226L** | **H274Y** | stable | 7.9 ± 0.1ºº | 7.3 ± 0.5ºº | 7.1 ± 0.2ºº | 7.5 ± 0.1*,ºº |
| **N158S/N248D** | **H274Y** | unstable | N/A | N/A | N/A | N/A |
| **Q226L/G228S** | **H274Y** | stable | 7.6 ± 0.1ºº | 7.5 ± 0.2*,ºº | 7.5 ± 0.2**,ºº | 7.0 ± 0.2ºº |
| **Q226L/N248D** | **H274Y** | unstable | N/A | N/A | N/A | N/A |
| **N158S/Q226L/N248D** | **H274Y** | stable | 7.5 ± 0.1ºº | 7.4 ± 0.6º | 6.7 ± 0.2ºº | 7.4 ± 0.4*,ºº |

^a^ Amino acid numbering is based on H3 HA and N2 NA (Nobusawa et al., 1991, Virology, 182, 475-485).

^b^ The genetic stability of recombinant H5N1 viruses was monitored by plaque assay and by sequencing of the HA and NA genes after transfection and after one passage in MDCK cells. Influenza virus was defined as genetically stable, if it was able to replicate efficiently in the cell line used, maintain a homogeneous plaque phenotype and did not contain additional subpopulations based on the sequence analysis of the HA and NA genes after one passage in MDCK cells. If different subpopulations were identified, those viruses were designated as unstable.

* *P* <0.05, ** *P* < 0.01 compared to wild-type rgVN1203 virus (one-way ANOVA performed for all viruses).

º *P* <0.05, ºº *P* <0.01 compared to H274Y virus (one-way ANOVA performed for viruses carrying the H274Y NA mutation).

N/A – not available due to instability of the H5N1 mutant virus.
